# Supplementary material for: Do behavioural risks cluster among college students in Chandigarh, India? Novel insights from a latent class analysis
Source: PLoS One. 2026 Jan 2;21(1):e0340072. doi: 10.1371/journal.pone.0340072 (PMC12758675; doi:10.1371/journal.pone.0340072)
Supplement: S1 File — (DOCX) [file pone.0340072.s001.docx]

**S1 File**

**Study questionnaire**

**Prevalence of Multiple Risk Behaviours among Youth in Chandigarh: A Cross-sectional Study**

| Date: ___________________ | Stream: _____________________ |
| --- | --- |

**Instructions**

- Do not write your name on this survey.
- Encircle the option which you feel is the most appropriate for you.
- Answer according to what you do in reality.
- The answers you give will be kept private and used for research purpose only. These will not affect your grades or career in any form.
- If you change your answer, please strikethrough the old option completely.

For example: □ ~~3rd year~~

- If you are not comfortable answering a question, just leave it blank.

| **Socio-demographic details and self-reported physical measurements** | | |
| --- | --- | --- |
| **1** | How old are you?  (Age in completed years) | □□ |
| **2** | What is your gender? | - Female - Male - Transgender |
| **3** | In which college year are you? | - - 1st year   - 2nd year   - 3rd year   - Final year   - Graduate or higher |
| **4** | How tall are you without your shoes on? (last recall value you can remember) | □ ft □ inches |
| **5** | How much do you weigh without your shoes on? (last recall value you can remember) | □□ kilograms |
| **Injury risks** | | |
| **6** | How often do you wear a seat belt while travelling in a car driven by someone else? | - Never - Rarely - Sometimes - Most of the time - Always |
| **7** | During the past 30 days, how many times did you ride in a car or other vehicle driven by someone who was under the influence of alcohol? | - 0 times - 1 time - 2 or 3 times - 4 or 5 times - 6 or more times |
| **8** | During the past 30 days, how many times did you drive a car or other vehicle under the influence of alcohol? | - I did not drive a car or other vehicle during the past 30 days - 0 times - 1 time - 2 or 3 times - 4 or 5 times - 6 or more times |
| **9** | During the past 30 days, on how many days did you text or e-mail or even talked on phone while driving a car or other vehicle? | - I did not drive a car or other vehicle during the past 30 days - 0 days - 1 or 2 days - 3 to 5 days - 6 to 9 days - 10 to 19 days - 20 to 29 days - All 30 days |
| **10** | During the past 12 months, how many times were you involved in a physical fight? | - 0 times - 1 time - 2 or 3 times - 4 or 5 times - 6 or 7 times - 8 or 9 times - 10 or 11 times - 12 or more times |
| **11** | During the past 12 months, how many times were you in a physical fight on campus property? | - 0 times - 1 time - 2 or 3 times - 4 or 5 times - 6 or 7 times - 8 or 9 times - 10 or 11 times - 12 or more times |
| **Victimisation** | | |
| **12** | During the past 12 months, how many times did someone you were dating or going out with physically hurt you on purpose? (Count such things as being hit, slammed into something, or injured with an object or weapon.) | - I did not date or go out with anyone during the past 12 months - 0 times - 1 time - 2 or 3 times - 4 or 5 times - 6 or more times |
| **13** | During the past 12 months, how many times did someone you were dating or going out with force you to do sexual things that you did not want to do? (Count such things as kissing, touching, or being physically forced to have sexual intercourse.) | - I did not date anyone during the past 12 months - 0 times - 1 time - 2 or 3 times - 4 or 5 times - 6 or more times |
| **14** | During the past 12 months, how many times did anyone force you to do sexual things that you did not want to do? (Count such things as kissing, touching, or being physically forced to have sexual intercourse.) | - 0 times - 1 time - 2 or 3 times - 4 or 5 times - 6 or more times |
| **15** | During the past 12 months, have you ever been bullied on campus property? | - Yes - No |
| **16** | During the past 12 months, were you electronically bullied? (Count being bullied through texting, Instagram, Facebook, or other social media.) | - Yes - No |
| **Depression and suicide risk** | | |
| **17** | During the past 12 months, did you ever feel so sad or hopeless almost every day for **two weeks or more in a row** that you stopped doing some usual activities? | - Yes - No |
| **18** | During the past 12 months, did you ever **seriously** consider attempting suicide? | - Yes - No |
| **19** | During the past 12 months, did you make a plan about how you would attempt suicide? | - Yes - No |
| **20** | During the past 12 months, how many times did you actually attempt suicide? | - 0 times - 1 time - 2 or 3 times - 4 or 5 times - 6 or more times |
| **21** | If you attempted suicide during the past 12 months, did any attempt result in an injury, poisoning, or overdose that had to be treated by a doctor or nurse? | - I did not attempt suicide during the past12 months - Yes - No |
| **Tobacco use** | | |
| **22** | Have you ever tried cigarette smoking, even one or two puffs? | - Yes - No |
| **23** | How old were you when you first tried cigarette smoking, even one or two puffs? | - I have never tried cigarette smoking. - 10 years old or younger - 10 to 13 years old - 13 to 17 years old - 17 years old or older |
| **24** | During the past 30 days, on how many days did you smoke cigarettes? | - 0 days - 1 or 2 days - 3 to 5 days - 6 to 9 days - 10 to 19 days - 20 to 29 days - All 30 days |
| **25** | During the past 30 days, on the days you smoked, how many cigarettes did you smoke per day? | - I did not smoke cigarettes during the past 30 days - Less than 1 cigaretteperday - 1 cigarette per day - 2to 5 cigarettes per day - 6 to 10 cigarettes perday - 11 to 20 cigarettes per day - More than 20 cigarettes per day |
| **26** | Have you ever used an electronic vapour product (e-cigarettes, e-cigars, e-pipes, e-hookahs and hookah pens)? | - Yes - No |
| **27** | Have you ever used a smokeless tobacco product like supari, gutka, pan, khaini, etc.? | - Yes - No |
| **28** | During the past 30 days, on how many days did you use smokeless tobacco products? | - 0 days - 1 or 2 days - 3 to 5 days - 6 to 9 days - 10 to 19 days - 20 to 29 days - All 30 days |
| **29** | During the past 30 days, how many packets of these tobacco products did you use? | - I did not use them - Less than 1 packet. - 1 packet per day. - 2-5 packets per day. - 5-10 packets per day. - More than 10 packets per day. |
| **30** | During the past 12 months, did you ever try to quit using all tobacco products, including cigarettes, cigars, smokeless tobacco, shisha or hookah tobacco, and electronic vapour products? | - I did not use any tobacco productsduring the past 12months - Yes - No |
| **Alcohol use** | | |
| **31** | Have you ever tried alcohol, even one or two sips? | - Yes - No |
| **32** | How old were you when you had your first drink of alcohol other than a few sips? | - I have never had a drink of alcohol other than a few sips - 10 years old or younger - 10 to 13 years old - 13 to 17 years old - 17 years old or older |
| **33** | During the past 30 days, on how many days did you have at least one drink of alcohol? | - 0 days - 1 or 2 days - 3 to 5 days - 6 to 9 days - 10 to 19 days - 20 to 29 days - All 30 days |
| **34** | During the past 30 days, how did you usually get the alcohol you drank? | - I did not drink alcohol in last 30 days - I bought it in a liquor store, - I bought it at a restaurant, bar, or club - I bought it at a public event such as a concert or sporting event - I gave someone else money to buy it for me - Someone gave it to me - I took it from a store or family member - I got it some other way |
| **35** | During the past 30 days, what is the largest number of alcoholic drinks you had in a row? | - I did not drink alcohol during the past 30 days - 1 or 2 drinks - 3 drinks - 4 drinks - 5 drinks - 6 or 7 drinks - 8 or 9 drinks - 10 or more drinks |
| **Use of other substances** | | |
| **36** | Have you ever used ganja, bhang, charas, joint or sulfa in your life? | - - Yes   - No |
| **37** | How old were you when you tried ganja, bhang, charas, joint or sulfa for the first time? | - - I have never tried marijuana   - 8 years old or younger   - 9 or 10 years old   - 11 or 12 years old   - 13 or 14 years old   - 15 or 16 years old   - 17 years old or older |
| **38** | During your life, how many times have you used ganja, bhang, charas, joint or sulfa in your life? | - - 0 times   - 1 or 2 times   - 3 to 9 times   - 10 to 19 times   - 20 to 39 times   - 40 or more times |
| **39** | During the past 30 days, how many times did you use ganja, bhang, charas, joint or sulfa? | - - 0 times   - 1 or 2 times   - 3 to 9 times   - 10 to 19 times   - 20 to 39 times   - 40 or more times |
| **40** | Have you ever used a drug (bhukki, smack, chitta, diluter, afeem, etc.) other than ganja, bhang, charas or joint? | - - Yes   - No |
| **41** | During your life, how many times have you used bhukki, smack, chitta, diluter, afeem, etc.? | - - 0 times   - 1 or 2 times   - 3 to 9 times   - 10 to 19 times   - 20 to 39 times   - 40 or more times |
| **42** | During your life, how many times have you sniffed diluter, glue, breathed the contents of aerosol spray cans, or inhaled any paints or sprays to get high? | - - 0 times   - 1 or 2 times   - 3 to 9 times   - 10 to 19 times   - 20 to 39 times |
| **43** | During your life, how many times have you used heroin (also called smack)? | - - 0 times   - 1 or 2 times   - 3 to 9 times   - 10 to 19 times   - 20 to 39 times   - 40 or more times |
| **44** | During your life, how many times have you used ecstasy (also called MDMA)? | - - 0 times   - 1 or 2 times   - 3 to 9 times   - 10 to 19 times   - 20 to 39 times   - 40 or more times |
| **45** | During your life, how many times have you taken prescription pain medicine and cough syrups without a doctor’s prescription or differently than how a doctor told you to use it? (Count drugs such as codeine, Corex, and Rexcoff.) | - - 0 times   - 1 or 2 times   - 3 to 9 times   - 10 to 19 times   - 20 to 39 times   - 40 or more times |
| **46** | During your life, how many times have you taken steroid pills or shots without a doctor’s prescription? | - - 0 times   - 1 or 2 times   - 3 to 9 times   - 10 to 19 times   - 20 to 39 times   - 40 or more times |
| **47** | During your life, how many times have you used a needle to inject any illegal drug into your body? | - - 0 times   - 1 time   - 2 or more times |
| **48** | During the past 12 months, has anyone offered, sold, or given you an illegal drug on or around college property? | - - Yes   - No |

| **Nutrition and diet** | | |
| --- | --- | --- |
| **49** | How do you describe your weight? | - - Very underweight   - Slightly underweight   - About the right weight   - Slightly overweight   - Very overweight |
| **50** | Which of the following are you trying to do about your weight? | - - Lose weight   - Gain weight   - Stay the same weight   - I am not trying anything |
| **51** | During the past 7 days, how many times did you drink 100% fruit juices such as orange juice, apple juice, or grape juice? (Do not count sports drinks, or other flavoured drinks.) | - - I did not drink 100% fruit juice during the past 7 days   - 1 to 3 times during the past 7 days   - 4 to 6 times during the past 7 days   - 1 time per day   - 2 times per day   - 3 times per day   - 4 or more times per day |
| **52** | During the past 7 days, how many times did you eat fruit? (Do not count fruit juice.) | - - I did not eat fruit during the past 7 days   - 1 to 3 times during the past 7 days   - 4 to 6 times during the past 7 days   - 1 time per day   - 2 times per day   - 3 times per day   - 4 or more times per day |
| **53** | During the past 7 days, how many times did you eat potatoes? (Do not count French-fries, fried potatoes, or potato chips.) | - - I did not eat potatoes during the past 7 days   - 1 to 3 times during the past 7 days   - 4 to 6 times during the past 7 days   - 1 time per day   - 2 times per day   - 3 times per day   - 4 or more times per day |
| **54** | During the past 7 days, how many times did you eat other vegetables? (Do not count green salad or potatoes.) | - - I did not eat other vegetables during the past 7 days   - 1 to 3 times during the past 7 days   - 4 to 6 times during the past 7 days   - 1 time per day   - 2 times per day   - 3 times per day   - 4 or more times per day |
| **55** | During the past 7 days, how many times did you drink a can, bottle, or glass of soda or pop, such as Coke, Pepsi, or Sprite? | - - I did not drink soda or pop during the past 7 days   - 1 to 3 times during the past 7 days   - 4 to 6 times during the past 7 days   - 1 time per day   - 2 times per day   - 3 times per day   - 4 or more times per day |
| **56** | During the past 7 days, how many glasses of milk did you drink? (Count the milk you drank in a glass or cup, from a carton, or with cereal. Count the half pint of milk served at school as equal to one glass.) | - - I did not drink milk during the past 7 days   - 1 to 3 glasses during the past 7 days   - 4 to 6 glasses during the past 7 days   - 1 glass per day   - 2 glasses per day   - 3 glasses per day   - 4 or more glasses per day |
| **57** | During the past 7 days, on how many days did you eat breakfast? | - - 0 days   - 1 day   - 2 days   - 3 days   - 4 days   - 5 days   - 6 days   - 7 days |
| **Physical activity, sedentary behaviour and sleep** | | |
| **58** | During the past 7 days, on how many days were you physically active for a total of at least 60 minutes per day? (Add up all the time you spent in any kind of physical activity that increased your heart rate and made you breathe hard some of the time.) | - 0 days - 1 day - 2 days - 3 days - 4 days - 5 days - 6 days - 7 days |
| **59** | During the past 7 days, on how many days did you do exercises to strengthen or tone your muscles, such as push-ups, sit-ups, or weight lifting? | - 0 days - 1 day - 2 days - 3 days - 4 days - 5 days - 6 days - 7 days |
| **60** | On an average school day, how many hours do you watch TV? | - I do not watch TV on an average school day - Less than 1 hour per day - 1 hour per day - 2 hours per day - 3 hours per day - 4 hours per day - 5 or more hours per day |
| **61** | On an average school day, how many hours do you play video or computer games or use a computer for something that is not school work? (Count time spent on things such as Xbox, PlayStation, an iPad or other tablet, a smartphone, texting, YouTube, Instagram, Facebook, or other social media.) | - I do not use a computer other than for school assignment. - Less than 1 hour per day - 1 hour per day - 2 hours per day - 3 hours per day - 4 hours per day - 5 or more hours per day |
| **62** | During the past 12 months, on how many sports teams did you play? (Count any teams run by your school or community groups.) | - 0 teams - 1 team - 2 teams - 3 or more teams |
| **63** | On an average school night, how many hours of sleep do you get? | - 4 or less hours - 5 hours - 6 hours - 7 hours - 8 hours - 9 hours - 10 or more hours |
| **64** | During the past 12 months, how would you describe your grades in school? | - Mostly A's - Mostly B's - Mostly C's - Mostly D's - Mostly F's - None of these grades - Not sure |
| **65** | Because of a physical, mental, or emotional problem, do you have serious difficulty concentrating, remembering, or making decisions? | - Yes - No |
| **Sexual behaviour** | | |
| **66** | Have you ever had sexual intercourse? | - Yes - No |
| **67** | How old were you when you had sexual  inter-course for the first time? | - I have never had sexual intercourse - 11 years old or younger - 12 years old - 13 years old - 14 years old - 15 years old - 16 years old - 17 years old or older |
| **68** | During your life, with how many people have you had sexual intercourse? | - I have never had sexual intercourse - 1 person - 2 people - 3 people - 4 people - 5 people - 6 or more people |
| **69** | During the past 3 months, with how many people did you have sexual intercourse? | - I have never had sexual intercourse - I have had sexual intercourse, but not during the past 3 months - 1 person - 2 people - 3 people - 4 people - 5 people - 6 or more people |
| **70** | Did you drink alcohol or use drugs before you had sexual intercourse the last time? | - I have never had sexual intercourse - Yes - No |
| **71** | The last time you had sexual intercourse; did you or your partner use a condom? | - I have never had sexual intercourse - Yes - No |
| **72** | The last time you had sexual intercourse, what one method did you or your partner use to prevent pregnancy? | - I have never had sexual intercourse - No method was used to prevent pregnancy - Birth control pills - Condoms - Withdrawal or some other method - Not sure |
| **73** | During your life, with whom have you had sexual contact? | - I have never had sexual contact - Females - Males - Females and males |
| **74** | Which of the following best describes you? | - Heterosexual (straight) - Gay or lesbian - Bisexual - Not sure |

**This ends the questionnaire.**

**Thank you very much for your help.**
