# Supplementary material for: Do behavioural risks cluster among college students in Chandigarh, India? Novel insights from a latent class analysis
Source: PLoS One. 2026 Jan 2;21(1):e0340072. doi: 10.1371/journal.pone.0340072 (PMC12758675; doi:10.1371/journal.pone.0340072)
Supplement: S2 File — (DOCX) [file pone.0340072.s002.docx]

**S2 File**

Prevalences of different behavioural risks among college students aged 18–22 years in Chandigarh, with 95% confidence intervals

| **Domain** | **Behavioural risk** | **Number with behavioural risk present** | **Number of individuals for whom valid responses were available** | **Prevalence (%)** | **95% CI for prevalence (%)** | |
| --- | --- | --- | --- | --- | --- | --- |
|  |  |  |  |  | **Lower limit** | **Upper limit** |
| **Injury risks** | Mobile use while driving in last 30 days | 138 | 746 | 18.5 | 15.8 | 21.5 |
|  | Involved in physical fight in last 12 months | 125 | 745 | 16.8 | 14.2 | 19.7 |
|  | Never or rarely uses seat belt | 68 | 752 | 9.0 | 7.1 | 11.4 |
|  | Driving under influence in last 30 days | 35 | 746 | 4.7 | 3.3 | 6.5 |
| **Victimisation** | Faced electronic bullying in last 12 months | 81 | 752 | 10.8 | 8.7 | 13.3 |
|  | Bullied on campus in last 12 months | 77 | 752 | 10.2 | 8.2 | 12.7 |
|  | Physically hurt while dating in the last 12 months | 29 | 729 | 4.0 | 2.7 | 5.7 |
|  | Faced sexual abuse in last 12 months | 15 | 729 | 2.1 | 1.2 | 3.5 |
| **Depression and suicide risk** | Felt sad or hopeless in last 12 months | 182 | 752 | 24.2 | 21.2 | 27.5 |
|  | Attempted suicide in last 12 months | 3 | 729 | 0.4 | 0.1 | 1.3 |
| **Substance use** | Current alcohol use | 144 | 726 | 19.8 | 17.0 | 23.0 |
|  | Binge drinking in last 30 days | 68 | 726 | 9.4 | 7.4 | 11.8 |
|  | Current cigarette smoking | 53 | 747 | 7.1 | 5.4 | 9.2 |
|  | Current cannabis use | 28 | 739 | 3.8 | 2.6 | 5.5 |
|  | Current smokeless tobacco use | 7 | 739 | 0.9 | 0.4 | 2.0 |
| **Nutrition and diet** | Did not eat vegetables twice daily in last week | 588 | 752 | 78.2 | 75.0 | 81.1 |
|  | Did not eat fruits once daily in last week | 527 | 752 | 70.1 | 66.6 | 73.3 |
|  | Drank aerated drinks every day in last week | 118 | 752 | 15.7 | 13.2 | 18.5 |
|  | Overweight/obese | 109 | 751 | 14.5 | 12.1 | 17.3 |
| **Physical activity, sedentary behaviour and sleep** | Insufficient physical activity | 450 | 752 | 59.8 | 56.2 | 63.4 |
|  | Muscle strengthening less than two days in last week | 445 | 752 | 59.2 | 55.6 | 62.7 |
|  | Average nighttime sleep <7 hours | 346 | 752 | 46.0 | 42.4 | 49.7 |
|  | Non-academic computer use 3 or more hours daily | 194 | 752 | 25.8 | 22.7 | 29.1 |
|  | TV viewing 3 or more hours daily | 118 | 752 | 15.7 | 13.2 | 18.5 |
| **Sexual behaviour** | Condom not used at last intercourse | 21 | 701 | 3.0 | 1.9 | 4.6 |
|  | Substance use before last intercourse | 20 | 701 | 2.9 | 1.8 | 4.5 |
|  | Four or more sexual partners in lifetime | 12 | 701 | 1.7 | 0.9 | 3.1 |
