# Supplementary material for: Do behavioural risks cluster among college students in Chandigarh, India? Novel insights from a latent class analysis
Source: PLoS One. 2026 Jan 2;21(1):e0340072. doi: 10.1371/journal.pone.0340072 (PMC12758675; doi:10.1371/journal.pone.0340072)
Supplement: S3 File — (DOCX) [file pone.0340072.s003.docx]

**S3 File**

Latent class model indices, for models with two to five latent classes

| **Number of classes** | **AIC** | **BIC** | **aBIC** | **Probability of membership in smallest latent class** | **Entropy** | **p-value for likelihood ratio test*** |
| --- | --- | --- | --- | --- | --- | --- |
| 2 | 13,469.3 | 13,728.1 | 13,550.3 | 16.7% | 0.930 | <0.001 |
| 3 | 13,356.7 | 13,749.7 | 13,479.7 | 15.5% | 0.818 | 0.036 |
| 4 | 13,256.1 | 13,783.0 | 13,421.0 | 5.4% | 0.916 | 0.016 |
| 5 | 13,204.2 | 13,865.2 | 13,411.2 | 3.2% | 0.887 | 0.366 |

* We used the Vuong–Lo–Mendell–Rubin likelihood ratio test to compare each model with the model that had one latent class less than it. E.g., the p-value for the two-class model was from the test comparing it with the one-class model.

Abbreviations: aBIC, sample size-adjusted Bayesian information criterion; AIC, Akaike information criterion; BIC, Bayesian information criterion.
