# Supplementary material for: Do behavioural risks cluster among college students in Chandigarh, India? Novel insights from a latent class analysis
Source: PLoS One. 2026 Jan 2;21(1):e0340072. doi: 10.1371/journal.pone.0340072 (PMC12758675; doi:10.1371/journal.pone.0340072)
Supplement: S4 File — (DOCX) [file pone.0340072.s004.docx]

**S4 file**

Fitted prevalences of different behavioural risks among college students aged 18–22 years in Chandigarh, by latent class

| **Behavioural risk** | **Fitted prevalence (%)** | | | |
| --- | --- | --- | --- | --- |
|  | Class 1 (multiple risks) | Class 2 (smoking and alcohol-related risks) | Class 3 (only dietary and physical activity risks) | Class 4 (victimisation and injury risks) |
| Never or rarely uses seat belt | 17.4 | 9.7 | 9.5 | 0.0 |
| Driving under influence in last 30 days | 48.2 | 1.8 | 0.4 | 1.0 |
| Mobile use while driving in last 30 days | 74.4 | 6.7 | 12.3 | 21.6 |
| Involved in physical fight in last 12 months | 51.1 | 0.0 | 12.9 | 25.0 |
| Bullied on campus in last 12 months | 31.1 | 0.0 | 8.0 | 13.5 |
| Faced electronic bullying in last 12 months | 23.1 | 9.6 | 7.6 | 21.6 |
| Physically hurt while dating in last 12 months | 31.3 | 4.9 | 1.6 | 0.0 |
| Faced sexual abuse in last 12 months | 8.5 | 0.0 | 0.8 | 6.4 |
| Felt sad or hopeless in last 12 months | 41.5 | 17.9 | 23.6 | 18.1 |
| Attempted suicide in last 12 months | 4.8 | 0.0 | 0.0 | 0.0 |
| Current cigarette smoking | 52.1 | 46.8 | 0.0 | 1.9 |
| Current smokeless tobacco use | 12.4 | 0.0 | 0.0 | 0.0 |
| Current alcohol use | 76.8 | 100.0 | 7.2 | 24.1 |
| Binge drinking in last 30 days | 45.4 | 100.0 | 0.0 | 4.1 |
| Current cannabis use | 40.3 | 0.0 | 0.5 | 0.0 |
| Overweight/obese | 17.7 | 26.8 | 11.2 | 27.0 |
| Drank aerated drinks every day in last week | 42.4 | 14.6 | 10.4 | 28.9 |
| Did not eat fruits once daily in last week | 53.9 | 76.4 | 75.4 | 46.8 |
| Did not eat vegetables twice daily in last week | 76.5 | 69.0 | 81.6 | 62.7 |
| Insufficient physical activity | 61.4 | 78.0 | 68.2 | 0.0 |
| Muscle strengthening less than two days in last week | 50.6 | 82.4 | 68.2 | 0.0 |
| TV viewing 3 or more hours daily | 10.7 | 13.8 | 13.4 | 34.3 |
| Non-academic computer use 3 or more hours daily | 28.6 | 9.9 | 27.4 | 21.1 |
| Average nighttime sleep <7 hours | 46.3 | 41.6 | 46.3 | 46.3 |
| Four or more sexual partners in lifetime | 16.1 | 11.0 | 0.0 | 0.0 |
| Substance use before last intercourse | 26.9 | 10.9 | 0.4 | 0.0 |
| Condom not used at last intercourse | 26.6 | 14.6 | 0.4 | 0.0 |
