# Supplementary material for: Do behavioural risks cluster among college students in Chandigarh, India? Novel insights from a latent class analysis
Source: PLoS One. 2026 Jan 2;21(1):e0340072. doi: 10.1371/journal.pone.0340072 (PMC12758675; doi:10.1371/journal.pone.0340072)
Supplement: S6 File — (DOCX) [file pone.0340072.s006.docx]

## R code used to calculate prevalence and its 95% confidence intervals for behavioural risks

# Importing data

d<-read.csv("S5 file.csv", header=TRUE)

## Checking how many variables each individual is contributing

d_orig<-d[,(setdiff(c(1:77),c(1,4,8)))]

# This gives the a data frame with only the original variables, barring sno, stream and bmi

resp<-rowSums(!is.na(d_orig)) # Number of questions for which each individual responded

table(resp) # Distribution of the variable 'resp'

median(replace(74-resp,resp==74,NA), na.rm=TRUE)

# Median number of questions missed, among those who had at least one missing

d[resp<=59,]

# Apparently these individuals have at least 15 missing variables!

# First recoding the variables

# In all cases, 1 is 'no', i.e. absence of risk factor.

# 2 is 'yes', i.e. presence of risk factor.

d$cbullied2<-d$cbullied+1 # Faced bullying in the campus

d$ebullied2<-d$ebullied+1 # Faced cyber bullying

d$sad2<-d$sad+1 # Felt sad or hopeless

d$seatbelt2 <- (d$seatbelt<3)+1 # Never or rarely wore seat belt

d$ddriveself2 <- (d$ddriveself>1 & d$ddriveself<6)+1

# Was driving under the influence of alcohol atleast once in last 30 days

d$mdrive2 <- (d$mdrive>1 & d$mdrive<8)+1 # Was using mobile while driving at least once in 30 days

d$fight2 <- (d$fight>1)+1 # Had a fight atleast once in the last 12 months

d$hurtdating2 <- (d$hurtdating>1 & d$hurtdating<6)+1 # Was physically hurt by the partner intentionally atleast once in the last 12 months.

d$sexabusedating2 <- (d$sexabusedating>1 & d$sexabusedating<6)+1 # Faced sexual abuse by the partner atleast once in the last 12 months.

d$asui2<-(d$asui>1)+1 # Attempted suicide atleast once in the last 12 months.

d$agesmoke2 <- (d$agesmoke<3)+1 # Started smoking before the age 13 years

d$smokedays2 <- (d$smokedays>1)+1 # Smoked in the last 30days

d$smokelessdays2 <- (d$smokelessdays>1)+1 # Used smokeless tobacco in the last 30days

d$agealc2<- (d$agealc<3)+1 # Started taking alcohol before the age of 13 years

d$alcdays2<-(d$alcdays>1)+1 # Used alcohol in the last 30days

d$bingedrinks2<- (d$bingedrinks!=8 & ((d$gender==1 & d$bingedrinks>3)|

(d$gender==2 & d$bingedrinks>2)))+1

# Binge drinking (5 drinks at a time for men, 4 for women)

d$agecann2<- (d$agecann<4)+1 # Started using marijuana before the age of 13 years

d$cannmonth2<- (d$cannmonth>1)+1 # Current Marijuana use

d$bmi2<- (d$bmi>=25)+1 # Overweight/ obese

d$break2<- (d$"break"==0)+1 # Skipped breakfast on all days in the last 7 days

d$fizzy2 <- (d$fizzy>2 & d$fizzy<7)+1 # Had atleast one cold drink per day in the last 7 days

d$fruit2 <- ((d$fruit<3)|(d$fruit==7))+1 # Did not have fruits at least once a day

d$veg2 <- ((d$veg<4)|(d$veg==7))+1 # Did not have vegetables at least twice a day

d$pa2 <- (d$pa<5)+1 # Did not exercise for atleast 5 days in the last 7 days

d$muscle2<- (d$muscle<2)+1 # Did not do muscular toning for atleast 2 days in the last 7 days

d$tv2<- (d$tv>3 & d$tv<7)+1 # Watched TV for 3 or more hours

d$comp2<- (d$comp>3 & d$comp<7)+1 # Used computer for 3 or more hours for non-school work

d$team2<- (d$team==1) + 1 # Did not participate in atleast 1 sports team

d$sleep2<- (d$sleep<4)+1 # Did not have minimum 7 hours of sleep

d$agesex2<- (d$agesex<4)+1 # Had sexual intercourse at age 13 or before

d$partner2<- (d$partner>3 & d$partner<7)+1 # Had 4 or more sexual partners during lifetime

d$subsex2<- (d$subsex==1)+1 # Had substance before having sexual intercourse

d$condom2<- (d$condom==0)+1 # Sexually active with last sex being condomless

# Making the prevalence table

prevtable<-data.frame(c(1:27),c(1:27),c(1:27),c(1:27), c(1:27), c(1:27))

colnames(prevtable)<-c("q","n","N","est","cil","ciu")

prevtable$q<-c("Never or rarely uses seat belt",

"Driving under influence in last 30 days",

"Mobile use while driving in last 30 days",

"Involved in physical fight in last 12 months",

"Bullied on campus in last 12 months",

"Faced electronic bullying in last 12 months",

"Physically hurt while dating in last 12 months",

"Faced sexual abuse in last 12 months",

"Felt sad or hopeless in last 12 months",

"Attempted suicide in last 12 months",

"Current cigarette smoking",

"Current smokeless tobacco use",

"Current alcohol use",

"Binge drinking in last 30 days",

"Current cannabis use",

"Overweight/obese",

"Drank aerated drinks every day in last week",

"Did not eat fruits once daily in last week",

"Did not eat vegetables twice daily in last week",

"Insufficient physical activity",

"Muscle strengthening less than two days in last week",

"TV viewing 3 or more hours daily ",

"Non-academic computer use 3 or more hours daily",

"Average nighttime sleep <7 hours",

"Four or more sexual partners in lifetime",

"Substance use before last intercourse",

"Condom not used at last intercourse")

prevtable$n<-c(sum(d$seatbelt2==2, na.rm=TRUE),

sum(d$ddriveself2==2, na.rm=TRUE),

sum(d$mdrive2==2, na.rm=TRUE),

sum(d$fight2==2, na.rm=TRUE),

sum(d$cbullied2==2, na.rm=TRUE),

sum(d$ebullied2==2, na.rm=TRUE),

sum(d$hurtdating2==2, na.rm=TRUE),

sum(d$sexabuse2==2, na.rm=TRUE),

sum(d$sad2==2, na.rm=TRUE),

sum(d$asui2==2, na.rm=TRUE),

sum(d$smokedays2==2, na.rm=TRUE),

sum(d$smokelessdays2==2, na.rm=TRUE),

sum(d$alcdays2==2, na.rm=TRUE),

sum(d$bingedrinks2==2, na.rm=TRUE),

sum(d$cannmonth2==2, na.rm=TRUE),

sum(d$bmi2==2, na.rm=TRUE),

sum(d$fizzy2==2, na.rm=TRUE),

sum(d$fruit2==2, na.rm=TRUE),

sum(d$veg2==2, na.rm=TRUE),

sum(d$pa2==2, na.rm=TRUE),

sum(d$muscle2==2, na.rm=TRUE),

sum(d$tv2==2, na.rm=TRUE),

sum(d$comp2==2, na.rm=TRUE),

sum(d$sleep2==2, na.rm=TRUE),

sum(d$partner2==2, na.rm=TRUE),

sum(d$subsex2==2, na.rm=TRUE),

sum(d$condom2==2, na.rm=TRUE))

prevtable$N<-c(sum(!is.na(d$seatbelt2)),

sum(!is.na(d$ddriveself2)),

sum(!is.na(d$mdrive2)),

sum(!is.na(d$fight2)),

sum(!is.na(d$cbullied2)),

sum(!is.na(d$ebullied2)),

sum(!is.na(d$hurtdating2)),

sum(!is.na(d$sexabuse2)),

sum(!is.na(d$sad2)),

sum(!is.na(d$asui2)),

sum(!is.na(d$smokedays2)),

sum(!is.na(d$smokelessdays2)),

sum(!is.na(d$alcdays2)),

sum(!is.na(d$bingedrinks2)),

sum(!is.na(d$cannmonth2)),

sum(!is.na(d$bmi2)),

sum(!is.na(d$fizzy2)),

sum(!is.na(d$fruit2)),

sum(!is.na(d$veg2)),

sum(!is.na(d$pa2)),

sum(!is.na(d$muscle2)),

sum(!is.na(d$tv2)),

sum(!is.na(d$comp2)),

sum(!is.na(d$sleep2)),

sum(!is.na(d$partner2)),

sum(!is.na(d$subsex2)),

sum(!is.na(d$condom2)))

for (i in 1:27){prevtable$est[i]<-100*(prop.test(prevtable$n[i],prevtable$N[i]))$estimate

prevtable[i,c("cil","ciu")]<-100*(prop.test(prevtable$n[i],prevtable$N[i]))$conf.int}

# Organising by domain and prevalence within domain

prevorder <- c(order(prevtable$est[1:4], decreasing=T), # Injury risks

4+order(prevtable$est[5:8], decreasing=T), # Victimisation

8+order(prevtable$est[9:10], decreasing=T), # Depression and suicide

10+order(prevtable$est[11:15], decreasing=T), # Substance use

15+order(prevtable$est[16:19], decreasing=T), # Nutrition and diet

19+order(prevtable$est[20:24], decreasing=T), # Physical activity and sleep

24+order(prevtable$est[25:27], decreasing=T)) # Sexual behaviour

# Graphing prevalence

par(mai=c(0.9,4,0.3,0.3),lwd=2)

barplot(prevtable[prevorder,"est"], names.arg=prevtable[prevorder,"q"], horiz=TRUE, las=1,

xlim=c(0,100), ylim=c(27,0), xlab="Prevalence (%)", xaxt="n",

main="Prevalence of behavioural risks among college students aged 18–22 years in Chandigarh",

width=1, space=0, cex.names=0.9,

col=c(rep("purple3",4),rep("steelblue3",4),rep("cyan3",2),rep("yellowgreen",5),rep("gold",4),

rep("orange",5),rep("orangered",3)),

border="white")

axis(side=1, at=seq(0, 100, 10))

arrows(prevtable[prevorder,"est"], seq(1,27)-0.5,

prevtable[prevorder,"cil"], seq(1,27)-0.5, angle=90, length=0.03, lwd=1)

arrows(prevtable[prevorder,"est"], seq(1,27)-0.5,

prevtable[prevorder,"ciu"], seq(1,27)-0.5, angle=90, length=0.03, lwd=1)

legend(x="topright", legend=c("Injury risks","Victimisation","Depression and suicide risk",

"Substance use","Nutrition and diet","Physical activity, sedentary behaviour and sleep",

"Sexual behaviour"),

fill=c("purple3","steelblue3","cyan3","yellowgreen","gold","orange","orangered"),

border="white", title="Domains")
