# Supplementary material for: Do behavioural risks cluster among college students in Chandigarh, India? Novel insights from a latent class analysis
Source: PLoS One. 2026 Jan 2;21(1):e0340072. doi: 10.1371/journal.pone.0340072 (PMC12758675; doi:10.1371/journal.pone.0340072)
Supplement: S7 File — (DOCX) [file pone.0340072.s007.docx]

! MPlus code used to perform latent class analysis for behavioural risks

! Data preparation: Remove header from the CSV file, replace all 'NA' with 999 and save in .dat format.

Title:

Latent Class Analysis.

Data:

File is data.dat;

Variable:

names = gender seatbelt2 ddriveself2 mdrive2 fight2 cbullied2 ebullied2

hurtdating2 sexabuse2 sad2 asui2 smokedays2 smokelessdays2 alcdays2

bingedrinks2 cannmonth2 bmi2 fizzy2 fruit2 veg2 pa2 muscle2 tv2 comp2 sleep2

partner2 subsex2 condom2;

usevariables = gender seatbelt2 ddriveself2 mdrive2 fight2 cbullied2 ebullied2

hurtdating2 sexabuse2 sad2 asui2 smokedays2 smokelessdays2 alcdays2

bingedrinks2 cannmonth2 bmi2 fizzy2 fruit2 veg2 pa2 muscle2 tv2 comp2 sleep2

partner2 subsex2 condom2;

categorical = seatbelt2 ddriveself2 mdrive2 fight2 cbullied2 ebullied2

hurtdating2 sexabuse2 sad2 asui2 smokedays2 smokelessdays2 alcdays2

bingedrinks2 cannmonth2 bmi2 fizzy2 fruit2 veg2 pa2 muscle2 tv2 comp2 sleep2

partner2 subsex2 condom2;

Missing are all (999);

classes = C(4);

Analysis:

Type=mixture;

Starts=10000 1000 100;

MODEL:

%OVERALL%

C ON gender;

Plot:

type is plot3;

series is seatbelt2 (1) ddriveself2 (2) mdrive2 (3) fight2 (4) cbullied2 (5)

ebullied2 (6) hurtdating2 (7) sexabuse2 (8) sad2 (9) asui2 (10)

smokedays2 (11) smokelessdays2 (12)

alcdays2 (13) bingedrinks2 (14) cannmonth2 (15) bmi2 (16)

fizzy2 (17) fruit2 (18)

veg2 (19) pa2 (20) muscle2 (21) tv2 (22) comp2 (23) sleep2

(24) partner2 (25) subsex2

(26) condom2 (27);

Savedata:

file is lca1_save.txt ;

save is cprob;

format is free;

output:

tech11 tech14;
