# Supplementary material for: Do behavioural risks cluster among college students in Chandigarh, India? Novel insights from a latent class analysis
Source: PLoS One. 2026 Jan 2;21(1):e0340072. doi: 10.1371/journal.pone.0340072 (PMC12758675; doi:10.1371/journal.pone.0340072)
Supplement: Attachment — Submitted filename: Response for reviewers.docx [file pone.0340072.s008.docx]

**Reply to comments from editor and reviewers**

Dear Editor,

Thank you for showing interest in our work. We are happy to receive insightful comments from the editor and reviewers. These comments have greatly helped us improve our manuscript. We have now substantially revised the analysis. We have also made several other revisions in the Title, Methods, Results and Discussion, to improve clarity. Below, we provide a point-by-point response to the editor and reviewer comments.

Regards,

The authors.

Note: To avoid confusion about the page numbers and line numbers in this response letter, please note that we have mentioned the page numbers and line numbers according to the final ‘track changes’ version of our revised manuscript.

**Editorial comments**

**1.Please ensure that your manuscript meets PLOS ONE's style requirements, including those for file naming. The PLOS ONE style templates can be found at**

[**https://journals.plos.org/plosone/s/file?id=wjVg/PLOSOne_formatting_sample_main_body.pdf**](https://journals.plos.org/plosone/s/file?id=wjVg/PLOSOne_formatting_sample_main_body.pdf) **and**

[**https://journals.plos.org/plosone/s/file?id=ba62/PLOSOne_formatting_sample_title_authors_affiliations.pdf**](https://track.editorialmanager.com/CL0/https:%2F%2Fjournals.plos.org%2Fplosone%2Fs%2Ffile%3Fid=ba62%2FPLOSOne_formatting_sample_title_authors_affiliations.pdf/1/010f0197458edae2-a7b2c5c3-83cb-433d-a41f-e0321415d338-000000/6dTYzZsH85O4HoCDIRxmtFXPfyGL7V0mXAStIEXKG2A=214)

Authors’ response:

We have now formatted the files and changed the file names, in accordance with the journal style.

- We have added page numbers at the bottom of each page in the main file.
- We have provided author names as a byline, with affiliations indicated by numbers in superscript.
- We have added heading levels (Heading 1 and Heading 2) as per the journal style guide.
- We have labelled figures as ‘Fig’, and named the figure files as ‘Fig1.tiff’, ‘Fig2.tiff’, and ‘Fig3.tiff’.
- We have named the supporting information files as ‘S1 File’, ‘S2 File’, so on.

**2. Please remove all personal information, ensure that the data shared are in accordance with participant consent, and re-upload a fully anonymized data set.**

**Note: spreadsheet columns with personal information must be removed and not hidden as all hidden columns will appear in the published file.**

**Additional guidance on preparing raw data for publication can be found in our Data Policy (**[**https://journals.plos.org/plosone/s/data-availability#loc-human-research-participant-data-and-other-sensitive-data**](https://journals.plos.org/plosone/s/data-availability#loc-human-research-participant-data-and-other-sensitive-data)**) and in the following article:** [**http://www.bmj.com/content/340/bmj.c181.long**](http://track.editorialmanager.com/CL0/http:%2F%2Fwww.bmj.com%2Fcontent%2F340%2Fbmj.c181.long/1/010f0197458edae2-a7b2c5c3-83cb-433d-a41f-e0321415d338-000000/Nbo-DvXh-XV356tjf8fx-xZunXqHo52o89kRZQiVOdg=214)**.**

Authors’ response:

The dataset we have uploaded does not contain any direct personal identifiers. It is anonymised, and does not contain the name or initials of any participant. We have now removed the variables ‘educational stream’ and ‘college year’ from the dataset. It is not possible to use the information remaining in our dataset to uniquely identify any individual. Our data sharing is in accordance with participant consent.

**3. We note that there is identifying data in the Supporting Information file <PLOS One Supplementary File S4.csv>. Due to the inclusion of these potentially identifying data, we have removed this file from your file inventory. Prior to sharing human research participant data, authors should consult with an ethics committee to ensure data are shared in accordance with participant consent and all applicable local laws.**

**Data sharing should never compromise participant privacy. It is therefore not appropriate to publicly share personally identifiable data on human research participants. The following are examples of data that should not be shared:**

**-Name, initials, physical address**

**-Ages more specific than whole numbers**

**-Internet protocol (IP) address**

**-Specific dates (birth dates, death dates, examination dates, etc.)**

**-Contact information such as phone number or email address**

**-Location data**

**-ID numbers that seem specific (long numbers, include initials, titled “Hospital ID”) rather than random (small numbers in numerical order)**

**Data that are not directly identifying may also be inappropriate to share, as in combination they can become identifying. For example, data collected from a small group of participants, vulnerable populations, or private groups should not be shared if they involve indirect identifiers (such as sex, ethnicity, location, etc.) that may risk the identification of study participants.**

**Additional guidance on preparing raw data for publication can be found in our Data Policy (**[**https://journals.plos.org/plosone/s/data-availability#loc-human-research-participant-data-and-other-sensitive-data**](https://journals.plos.org/plosone/s/data-availability#loc-human-research-participant-data-and-other-sensitive-data)**) and in the following article:** [**http://www.bmj.com/content/340/bmj.c181.long**](http://www.bmj.com/content/340/bmj.c181.long)**.**

**Please remove or anonymize all personal information (<specific identifying information in file to be removed>), ensure that the data shared are in accordance with participant consent, and re-upload a fully anonymized data set. Please note that spreadsheet columns with personal information must be removed and not hidden as all hidden columns will appear in the published file.**

Authors’ response:

We are committed to maintaining the privacy of study participants. The dataset we have uploaded does not contain any direct personal identifiers. It does not contain the name, initials, address or college name for any participant.

Also, we have now removed the variables ‘educational stream’ and ‘college year’ from the dataset (S5 File). These were indirect identifiers, and did not contribute to the main analysis. We have modified the Results accordingly.

P 10 lines 235–236

‘Among the 752 respondents, 268 (35.6%) identified as male, 484 (64.4%) identified as female, and none as transgender. The median age of the respondents was 19 years (IQR 19 to 21 years).’

Now, only two indirect identifiers remain in our dataset: age and gender. It is *not* possible to use the combined information from these two variables to uniquely identify any individual. These variables contributed to our results, and without them, readers may not be able to reproduce our findings.

Regarding the serial number column, we added it after entering the data. The serial number cannot be used to identify any individual.

We reiterate that our data sharing is in accordance with the protocol which our institute’s ethics committee approved. It is also in accordance with participant consent.

**4. We are unable to open your Supporting Information file PLOS One Supplementary File S5.R and PLOS One Supplementary File S6.R. Please kindly revise as necessary and re-upload.**

Authors’ response: We had earlier uploaded these files as R scripts. We are now uploading the files in Microsoft Word format, as ‘S6 File.docx’ and ‘S7 File.docx’.

S6 File: R code used to calculate overall prevalence and its 95% confidence intervals for behavioural risks

S7 File: MPlus code used to perform latent class analysis for behavioural risks

**Additional editor comments**

The author should strongly consider some of the following suggestion to improve the quality of the manuscript.

**1. Title: The study population (college going students from Chandigarh) cannot be considered as Indian youth. Do revise the title and objective to reflect the actual study population.**

Authors’ response: We agree. We have now modified the title to, ‘Do risky behaviours cluster among college students in Chandigarh, India? Novel insights from a latent class analysis’. We have also changed the short title to ‘Latent class analysis of behavioural risks among Chandigarh youth’.

We had already included the study population in the objectives as follows:

P 4 lines 93–94

‘Therefore, we planned this study to determine the prevalence of multiple behavioural risks among youth in a north Indian city, and identify clustering of risks within individuals, if any.’

**2. Ethical considerations: There are a few critical issues here. The manuscript elicited information on suicide ideation/attempts, unprotected sex, substance use (including illicit drugs), and experiences of violence or victimization. WHO ethical guidelines calls for a priori plans to refer participants who need mental-health services, and provide counseling or resource sheets, which are missing here.**

**In the research ethics frameworks, confidentiality has exceptions when a participant is at imminent risk of harm to self or others. Provide whether it was breached and did the findings of the study were shared with college administrations or concerned students to support targeted interventions?**

Authors’ response: We appreciate the editor’s attention to this very important point. Our study team had discussed this while planning the study. While collecting data with direct identifiers (such as names) would have allowed linkage with care, it would also have created concerns about confidentiality. It might also have influenced participants’ responses due to social desirability. Considering these factors, we decided to have anonymous data collection. We had mentioned this in the proposal that we submitted to the institute ethics committee, before starting the study. The institute ethics committee approved the proposal.

In terms of maintenance of anonymity, our methodology is the same as the original methodology used by the US CDC. See, for example:

Brener ND, Kann L, Shanklin S, Kinchen S, Eaton DK, Hawkins J, et al. Methodology of the Youth Risk Behavior Surveillance System — 2013. Morb Mortal Wkly Rep Recomm Rep. 2013;62: 1–23.

Underwood JM, Brener N, Thornton J, Harris WA, Bryan LN, Shanklin SL, et al. Overview and Methods for the Youth Risk Behavior Surveillance System - United States, 2019. MMWR Suppl. 2020 Aug 21;69(1):1-10. doi: 10.15585/mmwr.su6901a1.

We have now clarified these issues in the manuscript.

Study tool, p 7, lines 159–163

‘In total, the questionnaire comprised 74 questions. To maintain anonymity, it did not ask for the participant’s name or address. Most of the questions involved selecting responses from given options. Only the height and weight questions involved writing numerical digits in the data collection form. Hence, it was not possible to identify students by their handwriting.’

Ethical considerations, p 9, lines 216–219

‘Though the consent forms bore the signatures and names of participants, it was not possible to link them with the data collection forms which the participants submitted, thus ensuring anonymity. Given the anonymous nature of data collection, it was not possible for us to link participants with care if they reported behavioural risks (e.g., suicidal ideation or substance use).’

**3. Analysis: The manuscript statistical analysis had selected model based primarily on AIC, BIC, and interpretability of the fitted probabilities, they have not done any formal statistical tests, such as likelihood-ratio tests. While AIC and BIC are commonly used, the formal statistical tests can provide additional statistical evidence to support the chosen number of classes. The authors have not mention entropy value for the selected latent class model, which provides a key diagnostic for the quality of the latent class solution and the distinctiveness of the identified classes. Further having a sample size towards lower side and minimal differentiation of BIC curve between 4 and 5 classes, entropy values and likelihood-ratio tests are indeed indicated.**

Authors’ response: Thank you for this excellent suggestion. We have now revised the analysis. Since we could not obtain the entropy and likelihood ratio test p-values in R, we have now used MPlus for the latent class analysis.

Abstract, p 2

Lines 34–35: ‘We selected the most appropriate latent class model based on fitted probabilities, likelihood ratio tests, entropy, Akaike information criterion and Bayesian information criterion.’

Lines 37–38: ‘Latent class analysis identified four classes: multiple risks (8.8%), substance use and sexual risks (5.4%), low-risk (73.7%), and victimisation and injury risks (12.1%).’

Statistical analysis and reporting of results

p 8, lines 176–178

‘We entered the data into a spreadsheet, and then imported the spreadsheet to statistical analysis software. We did data processing, cleaning and prevalence estimation with R 4.3.0 (R Foundation for Statistical Computing, Vienna, Austria).’

P 9, lines 202–208

‘To identify clustering of risk factors, we performed latent class analysis using MPlus Version 8.4 (Muthen & Muthen). We included the 27 behavioural risks as binary variables, with gender as a covariate. We ran models with two to seven latent classes. We examined the fitted probabilities, entropy, p-values from the Vuong–Lo–Mendell–Rubin likelihood ratio test, the Akaike information criterion (AIC), Bayesian information criterion (BIC) and sample size-adjusted Bayesian information criterion (aBIC) for each model, to select the model with best performance.’

Results, p 11, lines 269–275

‘We selected the model with four classes as it was able to distinguish meaningful classes, had a relatively high entropy (0.916), and reasonably low AIC, BIC and aBIC (Figure 2 and S3 File). Also, the Vuong–Lo–Mendell–Rubin likelihood ratio test showed substantial improvement in fit up to four classes, but not on adding a fifth class (p=0.366 for five versus four classes). Figure 3 and S4 File show the pattern of item probabilities within these four classes. Some of the risks, namely nutritional risks, digital device use and sleep, did not differ appreciably across the classes. The other risks showed a distinctive pattern across classes.’

Figure 2: Assessment of latent class models for behavioural risks among youth in Chandigarh. Model fit was assessed using a. AIC, BIC and aBIC, and b. entropy, for models with different numbers of classes.

We have also added numerical values of these indices as supporting information:

S3 File: Latent class model indices, for models with two to five latent classes

| **Number of classes** | **AIC** | **BIC** | **aBIC** | **Probability of membership in smallest latent class** | **Entropy** | **p-value for likelihood ratio test*** |
| --- | --- | --- | --- | --- | --- | --- |
| 2 | 13,469.3 | 13,728.1 | 13,550.3 | 16.7% | 0.930 | <0.001 |
| 3 | 13,356.7 | 13,749.7 | 13,479.7 | 15.5% | 0.818 | 0.036 |
| 4 | 13,256.1 | 13,783.0 | 13,421.0 | 5.4% | 0.916 | 0.016 |
| 5 | 13,204.2 | 13,865.2 | 13,411.2 | 3.2% | 0.887 | 0.366 |

We have also revised Table 1, Figure 3 and S4 File accordingly.

**4. The Latent class analysis results description may be improved by incorporating a key component including describing each class with its size and % of total sample, high and low endorsement of key variables, and its label. Try to describe each class using this for better clarity and allowing the reader to understand the characteristics of each identified class.**

**ie. Class 4 (n=xxx; 6.1% of the sample) was characterized by high endorsement of current substance use (p = xx) and unprotected sex (p = xx) but low probabilities on other behavioral risks; accordingly, we labeled it the ‘Substance Use & Sexual Risks’ class.”**

Authors’ response: We have taken note of this suggestion. For clarity, we have now provided a detailed description of the latent classes in the Results.

P 12, lines 287–305

“Class 1 (estimated to constitute 8.8% of the population) was characterised by high endorsement of risks in almost all domains. These included driving under influence of alcohol (48.2%), mobile use while driving (74.4%), involvement in physical fights (51.1%), depressive symptoms (41.5%), cigarette smoking (52.1%), smokeless tobacco use (12.4%), alcohol use (76.8%), cannabis use (40.3%), substance use before last sexual intercourse (26.9%), and condomless sex (26.6%). Hence, we labelled this class as ‘multiple risks’.

Class 2 (5.4%) was marked by high endorsement of current cigarette smoking (46.8%), current alcohol use (100.0%), binge drinking (100.0%), four or more sexual partners in lifetime (11.0%), substance use before last sexual intercourse (10.9%), and condomless sex (14.6%). This class had low estimated prevalence of driving-related risks, physical violence and bully-victimisation. Accordingly, we labelled it as ‘substance use and sexual risks’.

In contrast, individuals in Class 3 (73.7%) had low estimated risk in most domains. Their prominent risks were diet, physical activity, digital device use, and sleep. Since these were uniformly high across almost all classes, we labelled Class 3 as ‘low risk’.

Finally, Class 4 (12.1%) was distinguished by high estimated prevalence of mobile use while driving (21.6%), involvement in physical fights (25.0%), bully-victimisation on campus (13.5%), and cyber bully-victimisation (21.6%). This was the only class with low prevalence of insufficient physical activity (0.0%) and inadequate muscle strengthening exercises (0.0%). We labelled this class as ‘victimisation and injury risks’.”

**Reviewers' comments:**

**Reviewer #1**

**1. Kindly mention, in detail, the details of division of colleges in different stratum and the basis adopted for it, since it has substantial impact on the generalizability of findings.**

Authors’ response: We thank the reviewer for this constructive comment.

At the time we conducted this study, Chandigarh had women’s colleges and co-educational colleges (but not a “men-only” college). From data available at that time, we had estimated that about one-sixth of the student population was enrolled in women’s colleges. Hence, we randomly selected one women’s college and five co-educational colleges. We have mentioned this in the manuscript:

P 6, lines 128–130:

‘Out of 32 government and private colleges in Chandigarh, six colleges were randomly selected—one women’s college and five co-educational colleges. This ratio was taken keeping in mind the relative numbers of students in these colleges.’

For selection within colleges, we did the selection with educational streams as strata, and sections as clusters within educational streams. We have now clarified this in the Methods:

P 6, lines 132–134

‘The colleges had three educational streams: Science, Arts and Commerce. For sampling within colleges, we considered the educational streams as strata. Within streams, students were divided into sections, which attended classes together. From each stream, we selected 3–4 sections randomly.’

**2. Also, was the data collection done anonymously, or some sort of identifier (like signed consent forms attached to questionnaire) was used. If anonymous, how was it maintained? Or if not anonymous, discuss the impact of social desirability bias in the limitations.**

Authors’ response: Our data collection was anonymous, and it was not possible to identify any participant from their responses. The consent form was not attached to the questionnaire, so it was not possible to identify individuals from their signed consent forms. We have now clarified this issue in the manuscript.

Study tool, p 7, lines 159–163

‘In total, the questionnaire comprised 74 questions. To maintain anonymity, it did not ask for the participant’s name or address. Most of the questions involved selecting responses from given options. Only the height and weight questions involved writing numerical digits in the data collection form. Hence, it was not possible to identify students by their handwriting.’

Ethical considerations, p 9, lines 216–219

‘Though the consent forms bore the signatures and names of participants, it was not possible to link them with the data collection forms which the participants submitted, thus ensuring anonymity. Given the anonymous nature of data collection, it was not possible for us to link participants with care if they reported behavioural risks (e.g., suicidal ideation or substance use).’

**3. Also, the do mention the specific modifications done to the standard YRBSS questionnaire, and the YRBSS module you used should be referenced. How was the relevant modifications suitable in the Indian context and its impact on international comparability could also be discussed.**

Authors’ response: Thank you for highlighting this. We have now added information on the version that we used, and the specific modifications that we made to the questionnaire:

Methods, p 6, lines 139–149,

‘This was an adapted version of the 2017 Youth Risk Behaviour Surveillance System (YRBSS) Standard High School questionnaire from the Centers for Disease Control and Prevention (CDC)[34], with a few changes to reduce respondent burden and make it relevant to the Indian scenario. The specific changes were as follows. We enquired about ‘college year’ instead of ‘grade’. We removed the items on race/ethnicity and gun violence, as we did not consider these sufficiently relevant in the local cultural context. In the section on ‘other tobacco products’, we retained only the items on smokeless tobacco products, as other tobacco types are extremely rare in this context. For smokeless tobacco products, we used terms in the local language. Similarly, for cannabis and cocaine, we used terms from the local language to improve comprehension. We removed the items on concussion, HIV, dental care and asthma to reduce respondent burden.’

These changes were deletions of some items that we found less relevant, and translations of a few terms. We did not add any new items to the questionnaire. The rest of the questions were identical to the original version. Therefore, we believe that our findings are internationally comparable. We have now added this to the Discussion:

P 19, lines 464–466

‘Though we removed some items and added local translations of a few terms, our findings are still internationally comparable, as the questionnaire was otherwise identical to the CDC YRBSS questionnaire.’

**Reviewer #2:**

**Line 1: The title should be according to journal guidelines.**

Authors’ response: Thank you for highlighting this. We have now modified the title, to clearly describe the study population. The revised title is, ‘Do risky behaviours cluster among college students in Chandigarh, India? Novel insights from a latent class analysis’.

**Line 2: The ‘period’ after the title should be deleted**

Authors’ response: Thank you for your attention to this detail, which we had overlooked. We have now removed the period from the end of the title.

**Line 3: The short title should be in title case format.**

Authors’ response: We have written the titles in sentence case, according to the journal submission guidelines: <https://journals.plos.org/plosone/s/submission-guidelines>.

**Lines 40 & 41: Should be adjusted as earlier suggested**

Authors’ response: We agree with this suggestion. We have now changed the title to accurately reflect the study population. We have also removed the period at the end of the title. The revised title is, ‘Do risky behaviours cluster among college students in Chandigarh, India? Novel insights from a latent class analysis’.

**Lines 70-73: You can rephrase as ‘In 2019, the Global Burden of Disease study estimated that unintentional injuries and transport accidents together accounted for a quarter of 72 the deaths in the 10–24 years age group in India, with self-harm and violence accounting for 73 another 17% of the deaths in this age group.**

Authors’ response: We agree that this sentence needs more clarity. The Global Burden of Disease findings we have cited pertained to the year 2019, but were published in 2021. Accordingly, we have reworded the text as follows.

P 3 lines 61–63,

‘Behavioural risks are an important contributor to morbidity and mortality among youth in India. This is elucidated by findings from the Global Burden of Disease study, published in 2021. In 2019, unintentional injuries and transport accidents together accounted for a quarter of the deaths in the 10–24 years age group in India, with self-harm and violence accounting for another 17% of the deaths in this age group.’

**Lines 86-87: “Similarly, a study in Bhubaneshwar found that almost half of college students engaged in physical activity ‘never’ or ‘occasionally’”. This statement is not clear. Kindly review for clarity.**

Authors’ response: Thank you for bringing this to our attention. We have now revised this sentence, to provide additional clarity, and ensure that it accurately represents the findings of the original study.

P 4 lines 80–81,

‘Similarly, a study in Bhubaneshwar found that almost half of college students did not follow a regular exercise routine.’

**Line 89: ‘Youth in India’ should be written as ‘Indian youth’**

Authors’ response: We agree that ‘Indian youth’ could be another way of writing this. However, use of nationality as an adjective implicitly assumes that nationality is an important part of people’s identity. We believe that it would be unfair on our part, as researchers, to make this assumption, especially when participants themselves have not said this explicitly. As epidemiologists, we wanted to emphasise on nationality as place of residence, accompanied by cultural and environmental influences unique to this region. Thus, we prefer to retain the form, ‘youth in India,’ consistently throughout the manuscript.

**Line 98: ‘Youth in India’ should be written as ‘Indian youths’**

Authors’ response: Thank you for this suggestion. From a scientific standpoint, we believe that nationality should be seen as a combination of place of residence, regional culture and environment, that shapes people’s behaviour. We would not like to use it as an adjective (implying it is an important part of someone’s identity) unless participants themselves say so. Hence, we have used, ‘youth in India’ consistently throughout the manuscript.

Also, since we meant to refer to a population, including both men and women, we have used ‘youth’ as an uncountable noun. ‘Youth/youths’ as a countable noun typically refers to individuals, especially boys or men.

**Lines 99- 100: Should be rephrased as ‘Therefore, this study aimed to determine the prevalence of multiple behavioural risks among youths in a north Indian city, and identify clustering of risks within individuals, if any’.**

Authors’ response: We agree that this could be another way of writing the objectives. We find the original version and the reviewer’s suggestion equivalent in terms of semantics. Hence, we have retained the original version of this sentence.

**Line 101: Since you did not investigate just one youth, then, the ‘youth’ should be written as ‘youths’.**

Authors’ response: We acknowledge the reviewer’s attention to detail. While writing this manuscript, we considered whether we should use ‘youth’ (as uncountable noun) or ‘youths’ (countable noun). We looked at some English dictionaries, which define the terms in this manner:

‘Youth’ as an uncountable noun refers to people as a group or population, and includes both boys/men and girls/women.

‘Youth/youths,’ as a countable noun, refers to people as individuals. This form is typically used for boys/men.

Sources:

Cambridge Dictionary: <https://dictionary.cambridge.org/dictionary/english/youth>

Merriam-Webster dictionary: <https://www.merriam-webster.com/dictionary/youth>

We meant to refer to a population rather than individuals. Also, we include both men and women. Hence, we have used ‘youth’ as an uncountable noun.

**Lines 103-106: ‘The city of Chandigarh is located in the northern part of India, and is notable for being a higher education hub, with a university and 26 colleges including over 80 departments.[28] Its student body comprises individuals hailing from northern India as well as other parts of the country.[29]’. This is describing the study location and should be moved to the ‘methods section’.**

Authors’ response: We agree that this describes the study setting rather than the objectives. We have now moved these two sentences to the Methods section, under ‘Study setting and design’ (p 5 lines 110–114).

**Lines 128-129: ‘This ratio was taken 129 keeping in mind the relative numbers of students in these colleges’. Kindly rephrase as ‘This proportion sampling technique was employed, given the relative student population in these colleges’.**

Authors’ response: We agree that this could be another way of writing. We find the original version and the reviewer’s suggestion equivalent in terms of semantics. Hence, we have retained the original version of this sentence.

**Lines 147-148: ‘One investigator’ cannot continue as ‘they’ in the following sentence. Kindly review for clarity.**

Authors’ response: Thank you for bringing this to our attention. The investigator who visited the schools prefers to use ‘he/him’ pronouns. Accordingly, we have changed the pronoun to ‘he’.

P 7 lines 165–169

‘After obtaining necessary permissions from the college principal, one of the investigators visited each college on a working day. He visited the classrooms where the selected sections of students studied, provided information about the nature and purposes of the study, and invited students to participate. He then distributed the questionnaire and a written consent form to all students in the classroom, and gave standardised instructions on filling the questionnaire.’

**Lines 173-173: ‘We defined ‘insufficient physical activity’ as being 174 physically active for at least 60 minutes a day for less than five days in the last week’. Please review this as it may be correct. Someone who had exercised for many hours for 4 days in a week cannot be considered inactive.**

Authors’ response: We agree that our method does not capture the exact amount of physical activity, in terms of intensity, frequency and duration. While such a measure would be ideal, it can only be obtained from tools designed specifically for this purpose, such as the Global Physical Activity Questionnaire. To minimise respondent burden, we used only one tool—the CDC YRBSS questionnaire—for this study. The item in this tool was, ‘During the past 7 days, on how many days were you physically active for a total of at least 60 minutes per day?’ It had options from 0 to 7 days. To maintain comparability with other studies, we did not make any changes in this item.

To dichotomise this item, we needed to fix a cutoff for number of days. We fixed the cutoff of 5 days/week as follows. The WHO guidelines on physical activity for adults (2020) recommend 150–300 minutes of moderate aerobic physical activity per week. We took the upper limit of this range, i.e. 300 minutes per week, and divided it by the number of minutes of physical activity per day (60) specified in the question. This came out to be 5 days. Hence, we considered that someone who responded with any number of days less than 5 had insufficient physical activity.

**Line 270: ‘Are like’ should be rephrased as ‘were similar to those of….’**

Authors’ response: We accept the reviewer’s suggestion and have changed the wording accordingly.

P 14 line 353

‘Our results were similar to those of NFHS–5’

**Line 272: Add respectively after this: ‘17% for 20–29 years.[11]’.**

Authors’ response: We have taken note of the reviewer’s suggestion. We believe that this sentence has sufficient clarity in its present form, and we would prefer to retain this form.

**Line 273: You can consider changing the verb ‘are’ to ‘may’ as your study design, a cross-sectional study and sample size, is not strong enough to make a national inference about Indian youths.**

Authors’ response: We agree that our study, given its design and study population, does not allow for inference at the national level. Hence, we do not make any such inference solely based on our study. This sentence in the Discussion (pp 14–15 lines 355–360) contains the interpretation of our findings in the context of the fifth round of the National Family Health Survey (NFHS–5). This nationally representative survey indeed shows that young adults in urban areas across the country, especially in Chandigarh and Delhi, have a high prevalence of obesity. Therefore, we have highlighted the similarity of our findings to the NFHS–5 findings, and provided a reference to the NFHS–5 national report alongside.

**Line 313: Please can you provide the age range of these young men for clearer comparison with your study participants.**

Authors’ response: Thank you for this suggestion. We have now added the age range covered by the primary study.

P 16 line 398,

‘In our study, 0.4% of students reported suicide attempts in the past 12 months, which is comparable to the study in Ballabgarh, Haryana, that found a prevalence of 0.8% among young men aged 18–24 years.[7]’

**Line 365: ‘Youth’ should be ‘youths’**

Authors’ response: We have taken due note of this suggestion from the reviewer. English dictionaries typically define ‘youth’ as having both a countable and an uncountable form:

‘Youth’ as an uncountable noun refers to people as a group or population, and includes both boys/men and girls/women.

‘Youth/youths,’ as a countable noun, refers to people as individuals. This form is most commonly used for boys/men.

Sources:

Cambridge Dictionary: <https://dictionary.cambridge.org/dictionary/english/youth>

Merriam-Webster dictionary: <https://www.merriam-webster.com/dictionary/youth>

Since we meant to refer to a population, including both men and women, we have used ‘youth’ as an uncountable noun.

**Line 375: ‘means’ should be ‘meant’.**

Authors’ response: Thank you for this suggestion. Since we are presenting an atemporal fact, i.e. what our study covers or implies, we consider the use of present tense (rather than past tense) appropriate here. Available scientific writing guides (such as <https://www.nature.com/scitable/topicpage/effective-writing-13815989/>) also support such use of the present tense.

**Line 376: ‘is’ should be ‘was’.**

Authors’ response: Thank you for this suggestion. Since we are presenting an atemporal fact, i.e. what our study covers or implies, we consider the use of present tense (rather than past tense) appropriate here. Available scientific writing guides (such as <https://www.nature.com/scitable/topicpage/effective-writing-13815989/>) also support such use of the present tense.

**Line 379: Since you are reporting a limitation of a study that was already conducted, I think it should be reported in past tense; ‘is’ should be ‘was’**

Authors’ response: Thank you for this suggestion. Since we are presenting an atemporal fact, i.e. what our study covers or implies, we consider the use of present tense (rather than past tense) appropriate here. Available scientific writing guides (such as <https://www.nature.com/scitable/topicpage/effective-writing-13815989/>) also support such use of the present tense.

**Line 380: ‘has’ should be ‘had’**

Authors’ response: Thank you for this suggestion. Since we are presenting a general truth (the high college enrolment rate in Chandigarh) rather than a finding of our study, we consider the use of present tense appropriate here. Available scientific writing guides (such as <https://www.nature.com/scitable/topicpage/effective-writing-13815989/>) also support such use of the present tense.

**Line 386: Kindly change ‘happened’ to ‘occurred’.**

Authors’ response: We accept the reviewer’s suggestion and have changed the text accordingly.

P 20 line 475

‘This could have occurred because of inability of participants to comprehend the questions, or respondent fatigue, as these items were towards the end.’

**Line 388: Change ‘is’ to ‘was’.**

Authors’ response: Thank you for this suggestion. We have now rephrased the sentence as follows.

P 20, lines 476–478

‘The use of self-reported height and weight, rather than anthropometric measurements, could bias our BMI estimates downwards, because of social desirability for thinness in this population.’

**Line 391: Change ‘youth’ to ‘youths’.**

Authors’ response: We thank the reviewer for this suggestion. English dictionaries typically define ‘youth’ as having both a countable and an uncountable form:

‘Youth’ as an uncountable noun refers to people as a group or population, and includes both boys/men and girls/women.

‘Youth/youths,’ as a countable noun, refers to people as individuals. This form is most commonly used for boys/men.

Sources:

Cambridge Dictionary: <https://dictionary.cambridge.org/dictionary/english/youth>

Merriam-Webster dictionary: <https://www.merriam-webster.com/dictionary/youth>

Since we meant to refer to a population, including both men and women, we have used ‘youth’ as an uncountable noun.
