# Supplementary material for: Do behavioural risks cluster among college students in Chandigarh, India? Novel insights from a latent class analysis
Source: PLoS One. 2026 Jan 2;21(1):e0340072. doi: 10.1371/journal.pone.0340072 (PMC12758675; doi:10.1371/journal.pone.0340072)
Supplement: Attachment — Submitted filename: Response to reviewers.docx [file pone.0340072.s010.docx]

**Reply to comments from editor and reviewers**

We thank the editor and reviewers for their time and expert inputs in this second round of review. Once again, our work has been enriched by the constructive comments of our peers. Below, we provide a point-by-point response to all the comments raised in this round of review. Please note that all page and line numbers refer to the locations in the track-changes version (not the clean version) of the manuscript.

With best regards,

The authors.

**Editorial comments**

**Your study addresses an important issue. Please clarify the definitions of risky behaviours, justify methodological choices, and refine interpretation of latent classes, particularly for low-prevalence behaviours and gender differences.**

Authors’ response

Thank you for your comments. We have now added a definition of ‘risky behaviours’ or ‘behavioural risks’ in the Introduction (p 4 lines 47–49). We have revised the Introduction and Methods section, to clarify our methods and our rationale for them. We have also renamed two of the latent classes, to better reflect their behavioural patterns. Class 2 is now labelled, ‘smoking, alcohol use and sexual risks’, while Class 3 is labelled, ‘only dietary and physical activity risks’. We have shortened our discussion of low-prevalence behaviours, and increased coverage of latent class analysis in the discussion. We have also toned down our conclusions on the mechanisms of gender differences, presenting them as future research directions instead.

**Reviewers' comments:**

**Reviewer #2: The author attended to the comments raised. The editor made a comprehensive review of the manuscript which made it more interesting and professional. Please note that extrapolation in science should always be done with caution.**

Authors’ response

We thank the reviewer for their inputs. We agree that extrapolation should be done cautiously. While drawing inferences from our findings, we have been cognisant of the limitations of our study (p 23 lines 452–466).

**Reviewer #3: Thank you for the opportunity to review the article “Do risky behaviours cluster among college students in Chandigarh, India? Novel insights from a latent class analysis”. This novel article addresses the prevalence of behavioral risks and identifies their clustering among young adults in Chandigarh, India. Here are some suggestions for the authors:**

**Regarding the formatting of the manuscript, I would adhere to traditional sections commonly found in a standard scientific manuscript, particularly in the method section, including participants, procedure, and statistical analysis. This way, the text is easier to follow.**

Authors’ response

We thank the reviewer for this suggestion. We agree that this could be another way of organising the manuscript. In line with journal guidelines, we have arranged our manuscript under the headings ‘Introduction’, ‘Methods’, ‘Results’ and ‘Discussion’. To maintain the flow of the Methods section, we have organised the Methods section in the following sub-headings.

Study setting and design

Study population and eligibility criteria

Study size and sampling strategy

Study tool

Data collection

Statistical analysis and reporting of results

Ethical considerations

**The data from lines 194-216 may be more visually appealing and understandable when displayed in a table.**

Authors’ response

We agree with the reviewer. Hence, we have presented the prevalence findings as a table in S2 File. To make the findings visually appealing and engaging, we have also presented them graphically in Figure 1.

**Overall, I believe the manuscript is well-written and addresses an interesting topic. Congratulations to the authors for their work.**

Authors’ response

Thank you very much for your interest in our work. We are happy to receive your helpful comments and appreciation.

**Reviewer #4: Strengths**

**• This study makes an important contribution by applying Latent Class Analysis (LCA) to explore risky behaviours among college students in Northern India. Given the limited data from South Asia, the findings are valuable for both public health and education policy.**

Authors’ response

Thank you for your positive assessment. We hope that our work will make a useful contribution to public health and education policy in South Asia and elsewhere.

**Major Comments**

**1. Class labelling (Lines 217, Supplementary File S4)**

**o The naming of latent classes could be reconsidered. For example, the “low risk” class still shows risks in nutrition and physical activity. Also, Class 1 appears to have higher sexual risk percentages than Class 2, which makes the current label “substance use and sexual risks” somewhat confusing. A clearer rationale or adjusted labels would improve interpretation.**

Authors’ response

We agree with the reviewer’s observation. We believe that renaming class 3 to ‘only dietary and physical activity risks’ will mitigate much of the confusion. So, we have made the following changes.

Abstract, lines 34–36:

‘Latent class analysis identified four classes: multiple risks (8.8%), smoking, alcohol use and sexual risks (5.4%), only dietary and physical activity risks (73.7%), and victimisation and injury risks (12.1%).’

Results, pp 14–15, lines 274–275

“Hence, we labelled Class 3 as ‘only dietary and physical activity risks’.”

We have also updated the class labels in Table 1 and S4 File.

Similarly, we have renamed Class 2 as ‘smoking, alcohol use and sexual risks’.

We agree that Class 1 has higher prevalence of sexual risks than Class 2. It is also important to recognise that, in any latent class analysis, the class names are short labels. They are meant for readers to quickly pick up patterns. The brevity of these labels means that some information will invariably be left out, and they cannot provide a comprehensive description of all the risk factors and their prevalences. This is why we have provided detailed numbers in the Results and S4 file, and a graph in Figure 3. We think that, with these details and the revised class labels, the readers will be able to get an in-depth interpretation of the patterns in the latent classes.

**2. Discussion alignment with the title (Lines 255, Discussion)**

**o Since the title emphasizes clustering through LCA, the Discussion would be stronger if it more clearly highlighted insights from the LCA results, with other findings used as supportive points.**

Authors’ response

We thank the reviewer for this suggestion. We have now greatly shortened the discussion on individual risk factors (pp 16–20). We have expanded the discussion on latent class analysis as follows.

P 20 lines 388–392

‘Similarly, the co-occurrence of victimisation, depression, and suicidal ideation, as seen in Class 1 in our study, has been noted in earlier work [62]. Indeed, these constructs might be causally linked, as shown by previous studies. For example, longitudinal studies show that cyber-bullying victimisation among adolescents correlates with subsequent depressive symptoms [63,64].’

P 22 lines 433–441

‘Studies among adolescents and young adults in other settings, such as the United States and Europe, show some parallels with our results. Dietary and physical activity risks tend to be quite common across classes [73]. Even where classes differ in the prevalence of unhealthy diet, sedentary behaviour and insufficient physical activity, inter-class differences are not marked [74–76] In contrast, substance use tends to show a much sharper variation across classes. The largest classes show low or no use of all substances, while a few small classes have very high prevalence for all substances [74,77]. Thus, our study, along with previous literature, shows that use of diverse substances such as alcohol, tobacco and cannabis tends to occur as highly concentrated syndemics.’

P 24 line 484–5

‘Another area of research could be tracking longitudinal changes in class membership over time [79].’

**3. Low-prevalence behaviours (S2 File)**

**o Behaviours such as sexual activity (3%) and suicide attempt (0.4%) have very low prevalence in this dataset. Discussion of these findings may need to be more cautious, or kept brief, to avoid overstating their implications.**

Authors’ response

We agree with the reviewer’s suggestion. We have now substantially shortened the discussion on sexual behaviours (p 18 lines 335–361) and suicide (pp 19–20 lines 363–378).

**4. Gender differences (Lines 345–349)**

**o The claim that men engage in more risky behaviours due to biological and social factors is interesting, but no supporting data were presented in this study. It may be better to frame this point as a possible future research direction rather than as a conclusion.**

Authors’ response

We agree with the reviewer on this point. While we have shown gender differences in risk clustering (Table 1), we have not demonstrated any biological or social mechanism for this difference. Accordingly, we have now toned down our conclusions in the Discussion. We have now presented them as possible explanations based on prior literature, and directions for future research.

Pp 20–21, lines 393–399

‘We observed a greater tendency of men to engage in risky behaviour. While we did not explore mechanisms for this difference, previous work shows that biological differences between men and women could play a role. The male hormone testosterone is known to promote aggression and risk-taking.[65] This might be reinforced by social norms which validate risky endeavours by men and discourage such endeavours by women.[66] Uncovering the relative contributions of nature and nurture to gendered risk-taking could be an idea for future research in India.’

**Reviewer #5: To provide context to the authors, I am a new reviewer brought on to review the manuscript between reviews. I have read both the responses to the editor and previous reviewers prior to reading the manuscript. The authors have addressed the reviewers’ and editor’s comments thoroughly. However, I believe there are still some issues in the reported analysis that require clarification.**

**Introduction: The introduction needs to be strengthened for better logical flow and coherence**

Authors’ response

We have tried to improve the flow of the Introduction based on the reviewer’s suggestions.

**1. In this manuscript, risky behavior is a key concept but its definition is unclear. The introduction lists different sets of behaviors in the first and second paragraphs, which are inconsistent and lack clear logic. For example, tobacco use and unhealthy diets appear once but are not discussed further, while it is questionable whether transport accidents should be classified as risky behaviors. The authors should provide a clear and consistent definition of risky behavior and ensure that the examples cited are logically connected and evidence-based.**

Authors’ response

We agree that defining risky behaviours is important for this study. Hence, we have now included the definition in the Introduction.

P 4 lines 47–52

‘While definitions vary, ‘risky behaviours’ or ‘behavioural risks’ are commonly conceptualised as avoidable actions, or omissions, of individuals which increase chances of adverse health outcomes for themselves or others [4]. Some examples of behavioural risks which often begin in youth are unsafe driving, tobacco use, alcohol use, drugs, violence, unsafe sexual behaviours and unhealthy diets.[5]’

We have also expanded the introduction to show more clearly the links of risky behaviours, including non-use of seat belts, tobacco use, and unhealthy diets, with health outcomes.

P 4 lines 59–64

‘In that year, alcohol use was found to be the most important behavioural risk for death in the 10–24 years age group, accounting for 2.6% of total deaths. Similarly, unsafe sex accounted for 0.9% of deaths and tobacco use for 0.3% of deaths.[7] Many of the deaths in transport accidents in India are attributable to behaviours such as non-use of seat belts and helmets [8]. Unhealthy diets and insufficient physical activity, when established during youth and persistent in the long term, show their effects late in the life course, in the form of non-communicable diseases like cardiovascular disease [9].’

**2. Sexual risk behavior plays an important role in the results, where Class 2 is labeled as substance use and sexual risks, and it is also extensively discussed in the discussion section. However, the introduction provides very limited description of sexual risk behavior. Moreover, there is a substantial body of literature on sexual risk among college students, yet none of these studies are cited. The introduction would be strengthened by a clearer discussion of sexual risk behavior and by incorporating relevant evidence from existing research.**

Authors’ response

We thank the reviewer for highlighting this. In the Introduction section, we have now elaborated on sexual risks among youth in India, based on a more extensive review of literature.

P 5 lines 76–84

‘Sexual activity in youth is rare in India, as compared to other countries [17]. Yet, among those who are sexually active, the prevalence of condomless sex may be quite high. This is particularly true for boys and young men. A study conducted in Chandigarh in 2020 found that nearly two-thirds of sexually active college students had unprotected sex [18]. Another study among men aged 18–24 in Ballabgarh, Haryana, found that almost one-third of those sexually active had engaged in condomless sex [10]. Use of psychoactive substances before sex is another concern, as it impairs judgement and decreases condom use [19]. There are limited data on this practice among youth in India [20].’

**3. The study focuses on college students, yet the introduction primarily discusses youth, and only mentions college students in the final paragraph. This issue was raised in previous rounds of review, but it does not appear to have been adequately addressed. The rationale for focusing specifically on college students is not sufficiently convincing. For instance, the authors suggest that college students have increased autonomy because they are less controlled by parents, but this characteristic may also apply to young people who do not attend college. The introduction would be strengthened by providing evidence that risk behaviors are particularly prevalent among college students compared to their non-college peers.**

Authors’ response

We thank the reviewer for bringing this to our attention. We have revised the Introduction accordingly. We have now elaborated on our rationale for including college students in this study.

P 6 lines 98–105

‘We focused on youth attending colleges for higher education. We did this for several reasons. One, colleges were sites where we could readily access eligible individuals, using the limited resources that we had for this study. Two, colleges are an important avenue for youth health promotion programmes [33,34]. Three, in the Indian context, higher education is a critical phase where the increased autonomy from parental control means that a person is likely to adopt unsafe behaviours.[35,36] College students may show greater prevalence of behavioural risks, such as binge drinking, than their non-college peers [37].’

**Methods**

**4. Regarding the sample size calculation, it is unclear why the authors used smokeless tobacco use as the reference. This study includes a range of risky behaviors, not just tobacco use. It may be more appropriate to base the calculation on the prevalence of risky behaviors among college students reported in previous studies, for example using the median rate across different behaviors.**

Authors’ response

We thank the reviewer for taking note of this. For estimating a prevalence *p* with relative error *r* and confidence level 100(1−*α*)%, the required sample size is (*Z*^2)*p*(1−*p*)/((*rp*)^2), where *Z* is the standard normal deviate corresponding to the chosen *α*. It can be shown that, for a constant *r*, this sample size decreases monotonically with increasing *p*. In other words, for a given relative error, outcomes with lower prevalence (i.e., rare outcomes) need greater sample size for prevalence estimation. We chose smokeless tobacco use as its anticipated prevalence, 10.8%, was on the lower side among the behaviours we were interested in. With this prevalence and 20% relative error, we got a sample size of 793.

An interesting question would be, is this sample size sufficient for the other risks too? With this sample size of 793, we estimated reasonable relative errors for common behavioural risks. For example, with this sample size, for overweight (anticipated prevalence 31.1%), we would have a relative error of 10.4%. On the other hand, for the rarer risks, we anticipated worse precision. For example, for condomless sex (anticipated prevalence 2.6%), we anticipated the relative error to be as high as 42.6%.

Table: Absolute and relative errors for estimating prevalence of different behavioural risks, assuming a final sample size of 793, and prevalence from prior literature

| Behavioural risk factor | Data source for anticipated prevalence | Anticipated prevalence | Absolute error | Relative error |
| --- | --- | --- | --- | --- |
| Mobile use while driving in last 30 days | Kulkarni V, Kanchan T, Palanivel C, Papanna MK, Kumar N, Unnikrishnan B. J Forensic Leg Med. 2013 May;20(4):226-9. | 20.5% | 2.8% | 13.7% |
| Driving under influence in last 30 days | Kulkarni V, Kanchan T, Palanivel C, Papanna MK, Kumar N, Unnikrishnan B. J Forensic Leg Med. 2013 May;20(4):226-9. | 25.2% | 3.0% | 12.0% |
| Sexual abuse victimisation | Ts J, Rani A, Menon PG, Cr J, M R, Jose V, Ks R, Kishore A, K T, B SN. Asian J Psychiatr. 2017 Aug;28:28-31. | 10.4% | 2.1% | 20.4% |
| Depressive symptoms in last 12 months | Singh M, Goel NK, Sharma MK, Bakshi RK. National Journal of Community Medicine. 2017 Nov 30;8(11):666-671. | 59.2% | 3.4% | 5.8% |
| Suicide attempt in last 12 months | Nath Y, Paris J, Thombs B, Kirmayer L. Int J Soc Psychiatry. 2012 Jul;58(4):393-9. | 4.0% | 1.4% | 34.1% |
| Current cannabis use | Gupta S, Sarpal SS, Kumar D, Kaur T, Arora S. J Clin Diagn Res. 2013;7: 1632–1636. | 3.9% | 1.3% | 34.6% |
| Current smokeless tobacco use | Global Adult Tobacco Survey, India 2016-17 report. (15–24 years’ age group) | 10.8% | 2.2% | 20.0% |
| Current cigarette smoking | Global Adult Tobacco Survey, India 2016-17 report. (15–24 years’ age group) | 4.0% | 1.4% | 34.1% |
| Current alcohol use | National Family Health Survey, 5^th^ round 2015–16, India report. (15–19 years’ age group) | 4.7% | 1.5% | 31.3% |
| Overweight | Raj S, Senjam SS, Singh A. Int J Prev Med. 2013;4: 1189–1193. | 31.1% | 3.2% | 10.4% |
| Insufficient physical activity | Raj S, Senjam SS, Singh A. Int J Prev Med. 2013;4: 1189–1193. | 28.6% | 3.1% | 11.0% |
| Insufficient intake of fruits | Raj S, Senjam SS, Singh A. Int J Prev Med. 2013;4: 1189–1193. | 46.9% | 3.5% | 7.4% |
| Sexually active, with condom not used at last sexual intercourse | National Family Health Survey, 5^th^ round 2015–16, India report. (Used national-level values for never-married, urban individuals aged 15–24 years) | 2.6% | 1.1% | 42.6% |

We have now acknowledged this as a limitation.

P 23, lines 452–454

‘One limitation of this study is the study size. While it was sufficient for estimating prevalence of common risks (prevalence >10%) and latent class modelling, it was not sufficient for estimating prevalence of rarer risks such as suicide attempts and unsafe sexual behaviours.’

**5. The inclusion criteria restrict participants to ages 18–22, but the rationale for this age range is unclear. In the introduction, the cited evidence refers to individuals under 24. Are college students in India generally under 22? If some students are older due to gaps or delayed enrollment (e.g., 22–24 years), will they be excluded? The authors should clarify the reason for this age restriction and justify why older college students are not included.**

Authors’ response

We thank the reviewer for highlighting this. Indeed, in the setting where we recruited students, most students enrol in colleges around 18 years of age and have four-year courses. Thus, most college students are in the 18–22 years age group. We agree that, because of delayed enrolment, gaps or inability to complete their course in four years, a small minority of college students could be more than 22 years of age. To ensure a homogeneous population and for ease of understanding, we capped the age limit in our eligibility criteria at 22 years. We acknowledge that this could have implications for transportability of findings beyond this age group.

Strengths and limitations, p 24 lines 465–466

‘Finally, extrapolation of our findings to youth less than 18 years of age or greater than 22 years of age should be done with caution.’

**6. line 166, the authors mention that they listed 27 important behavioral risks. It is unclear what the basis for this selection is. Are these all behavioral risks included in the YRBSS, or were they selected by the authors as deemed important? If the latter, a supporting reference should be provided. If the YRBSS includes only these 27 risks, this should be clearly stated in the Study Tool section.**

Authors’ response

The reviewer has highlighted an important question which many readers might raise. Our answer is, no, these were not all the risks which the YRBSS tool had. We had shortlisted some behavioural risks, keeping in mind our plan for latent class modelling, public health importance of the risks, overlap between risks and observed prevalence. We have now explained this in the Methods section.

P 10 lines 181–190

“We then listed 27 behavioural risks for which we had data available, and which we considered sufficiently important, based on the literature on health risks among youth in India [7]., While shortlisting these 27 risks, we tried to ensure diversity in terms of the items covered, while maintaining parsimony of items for latent class modelling. Accordingly, we omitted items which we found extremely rare (e.g., solvent and heroin use) or of uncertain importance for public health (e.g., dairy consumption). Also, where multiple items measured nearly identical constructs, we included only one. For example, ‘faced sexual abuse during dating in the last 12 months’ was highly correlated with ‘faced sexual abuse in the last 12 months,’ so we kept the latter only.”

**7. Supplementary Table S2 shows that the 27 behavioral risks are grouped into seven categories: Injury risks, Victimization, Depression and suicide risk, Substance use, Nutrition and diet, Physical activity, sedentary behavior and sleep, and Sexual behavior. The authors should describe this categorization in the Study Tool section. Without this explanation, readers may find it difficult to understand the composition and classification of the 27 important behavioral risks.**

Authors’ response

Thank you for highlighting this. We have now added a description of this categorisation in the Methods, for additional clarity.

pp 10–11, lines 190–194

‘For ease of interpretation, we also organised them into seven broad domains: injury risks (four items), victimisation (four items), depression and suicide risk (two items), substance use (five items), nutrition and diet (four items), physical activity, sedentary behavior and sleep (five items), and sexual behavior (three items).’

**Results**

**8. The label for Class 2 (substance use and sexual risks) may be misleading. As shown in Supplementary File S4, Class 2 is primarily characterized by alcohol use and smoking, whereas cannabis use and tobacco use are both zero. In fact, cannabis and tobacco use are significantly higher in the Multiple Risk group. Naming Class 2 as substance use and sexual risks could therefore give a misleading impression of its behavioral profile.**

Authors’ response

We agree with the reviewer’s observation. To give a more accurate impression of the behavioural pattern in Class 2, we have now labelled this class as ‘smoking, alcohol use and sexual risks’.

Abstract, lines 34–36:

‘Latent class analysis identified four classes: multiple risks (8.8%), smoking, alcohol use and sexual risks (5.4%), only dietary and physical activity risks (73.7%), and victimisation and injury risks (12.1%).’

Results, p 14, line 271

“Accordingly, we labelled it as ‘smoking, alcohol use and sexual risks’.”

We have also updated the class labels in Table 1 and S4 File.

**9. In Class 2, 10.9% of participants reported substance use before their last intercourse, yet the data show that cannabis use and tobacco use are both zero. This appears inconsistent. If substance use before sexual activity does not include cannabis and tobacco, the assessment of substance use may be incomplete, potentially overlooking important behaviors.**

Authors’ response

We appreciate the reviewer’s keen observation. Yet, we see no inconsistency here. The question in our data collection tool was, ‘Did you drink alcohol or use drugs before you had sexual intercourse the last time?’ Thus, ‘substance use before sex’ includes use of alcohol and other drugs before sex. Our finding shows that, among the psychoactive substances that people in Class 2 used before sex, alcohol was the predominant substance (indeed, prevalence of current alcohol use in this class was 100%).
