# Supplementary material for: Do behavioural risks cluster among college students in Chandigarh, India? Novel insights from a latent class analysis
Source: PLoS One. 2026 Jan 2;21(1):e0340072. doi: 10.1371/journal.pone.0340072 (PMC12758675; doi:10.1371/journal.pone.0340072)
Supplement: Attachment — Submitted filename: Response_to_reviewers_auresp_3.docx [file pone.0340072.s011.docx]

**Reply to comments from editor and reviewers**

Dear Editor,

Thank you for giving us the opportunity to improve our work once again. We have made some changes to the manuscript, in line with editor and reviewer comments.

With best regards,

Corresponding author.

**Editor Comments**

**One of reviewers requested additional work for the label of class and consistent use of terminology. Please revise.**

Author response

We have revised the class labels, and used the term ‘behavioural risks’ consistently throughout the manuscript. We have also changed the title to ‘Do behavioural risks cluster among college students in Chandigarh, India? Novel insights from a latent class analysis’ as suggested by Reviewer #4.

**Reviewer #4**

**Thank you for submitting the revised version of your manuscript. The study remains important in the context of public health and education policy, and the analytical approach is appropriate. The structure and clarity have improved compared with the previous version. However, one previously raised issue has not yet been adequately addressed, and one new point requires clarification and revision.**

**1. Class Labelling (Lines 217; Supplementary File S4) (Previously raised in Round 1 but not sufficiently resolved) Class 2 continues to be labelled “smoking, alcohol use and sexual risks,” which remains inconsistent with both the statistical results and conceptual interpretation.**

**a. Very low prevalence of sexual behaviour variables**

**According to the S2 File, variables related to sexual behaviour have extremely low prevalence, with the highest frequency being 21 participants (about 3%). Such a small subsample cannot provide stable class estimation; low-prevalence indicators often yield unstable parameter estimates in LCA and therefore should not define a class.**

Author response

We agree that items with very low or very high overall probability have unstable estimates in latent class analysis. Indeed, some risk factors in our study, such as suicide attempts and unsafe sexual behaviours, had low prevalences.

**b. Fitted prevalence patterns do not support the label**

**The S4 File shows that sexual-risk indicators are actually higher in Class 1 (multiple risks) than in Class 2. For example:- Condom not used at last intercourse: Class 1 = 26.6%, Class 2 = 14.6%- Four or more sexual partners in lifetime: Class 1 = 16.1%, Class 2 = 11.0%These results indicate that sexual-risk behaviours are characteristic of Class 1, not Class 2. Including “sexual risks” in the Class 2 label may therefore be misleading.**

Author response

We agree that Class 1 has a higher prevalence of sexual risks than Class 2. However, it is also worth noting that the prevalence of these risk factors in Class 2 is much higher than that in Classes 3 and 4. E.g., ‘four or more sexual partners in lifetime’, Class 2: 11.0%, Classes 3 and 4: both 0.0%. Similarly, for ‘condom not used at last intercourse’: Class 2: 14.6%, Class 3: 0.4%, Class 4: 0.0%. This shows a distinct pattern of sexual risks in Class 2. (Whether these prevalence estimates are reliable is a separate issue, covered in point a. above.)

**Recommendation: Please reconsider the naming of Class 2 to reflect its dominant behavioural profile more accurately—for example:- “smoking and alcohol-related risks”, or- “substance use–dominant risks.” Adding a brief statement in the Methods or Results section explaining the rationale for final class names (e.g., theoretical justification, indicator strength, or item-response probabilities) would also improve transparency.**

Author response

Overall, we agree with the reviewer’s recommendation, particularly in light of point a. (low-prevalence behaviours should not define a class). Hence, we have labelled class 2 as ‘smoking and alcohol-related risks’ throughout the manuscript.

Abstract, p 2 lines 34–36:

‘Latent class analysis identified four classes: multiple risks (8.8%), smoking and alcohol-related risks (5.4%), only dietary and physical activity risks (73.7%), and victimisation and injury risks (12.1%).’

Results, p 14 line 273

“Accordingly, we labelled it as ‘smoking and alcohol-related risks’.”

We have also revised the label for Class 2 in Table 1 and S4 File.

We have now provided a brief description of the rationale and process of naming classes in the Methods.

pp 11–12, lines 209–213

‘We also assigned short, descriptive labels to each class, based on the model estimates. We labelled classes according to the behavioural risk domains which showed greatest within-class fitted prevalence. Though we had included rare risk factors (e.g., suicide attempts, sexual risks) in the models, we avoided including them in class labels, as their parameter estimates could be statistically unstable.’

**2. Terminology Consistency (New observation in Round 2)**

**The manuscript alternates between “risky behaviours” and “behavioural risks.” Although the two expressions are related, consistent terminology is necessary for clarity and precision. Given that this study adopts an epidemiological and risk-modelling perspective, “behavioural risks” is the more technically accurate term because it frames behaviours as risk factors rather than inherently risky actions.**

**Recommendation: Please standardise the terminology throughout the manuscript—particularly in- the title and abstract,- figure and table captions, and- the main text and Discussion.**

Author response

We agree with the reviewer’s recommendation. We have now used the term ‘behavioural risks’ consistently throughout the manuscript.

**Maintain British spelling (e.g., behaviour, behavioural) consistently across the entire manuscript.**

Author response

We thank the reviewer for bringing this to our attention. We have now used British spelling (e.g., ‘behavioural’ rather than ‘behavioral’) throughout the manuscript text. While referring to the CDC YRBSS, we have kept the spelling as the original American ‘Behavior’. Similarly, where American spellings come up in the References, we have kept them unchanged.

**Reviewer #5**

**I have reviewed the authors' responses to my revision requests, and they have satisfactorily addressed the issues.**

Author response

We thank the reviewer for their constructive inputs, which helped us revise this manuscript.
