# Supplementary material for: Do behavioural risks cluster among college students in Chandigarh, India? Novel insights from a latent class analysis
Source: PLoS One. 2026 Jan 2;21(1):e0340072. doi: 10.1371/journal.pone.0340072 (PMC12758675; doi:10.1371/journal.pone.0340072)
Supplement: Attachment — Submitted filename: reviewer comment (PONE-D-25-12897R1) .pdf [file pone.0340072.s009.pdf]

To provide context to the authors, I am a new reviewer brought on to review the manuscript between reviews. I have read both the responses to the editor and previous reviewers prior to reading the manuscript. The authors have addressed the reviewers' and editor's comments thoroughly. However, I believe there are still some issues in the reported analysis that require clarification.

Introduction: The introduction needs to be strengthened for better logical flow and coherence

1. In this manuscript, risky behavior is a key concept but its definition is unclear. The introduction lists different sets of behaviors in the first and second paragraphs, which are inconsistent and lack clear logic. For example, tobacco use and unhealthy diets appear once but are not discussed further, while it is questionable whether transport accidents should be classified as risky behaviors. The authors should provide a clear and consistent definition of risky behavior and ensure that the examples cited are logically connected and evidence-based.

2. Sexual risk behavior plays an important role in the results, where Class 2 is labeled as substance use and sexual risks, and it is also extensively discussed in the discussion section. However, the introduction provides very limited description of sexual risk behavior. Moreover, there is a substantial body of literature on sexual risk among college students, yet none of these studies are cited. The introduction would be strengthened by a clearer discussion of sexual risk behavior and by incorporating relevant evidence from existing research.

3.. The study focuses on college students, yet the introduction primarily discusses youth, and only mentions college students in the final paragraph. This issue was raised in previous rounds of review, but it does not appear to have been adequately addressed. The rationale for focusing specifically on college students is not sufficiently convincing. For instance, the authors suggest that college students have increased autonomy because they are less controlled by parents, but this characteristic may also apply to young people who do not attend college. The introduction would be strengthened by providing evidence that risk behaviors are particularly prevalent among college students compared to their non-college peers.

Methods

4. Regarding the sample size calculation, it is unclear why the authors used smokeless tobacco use as the reference. This study includes a range of risky behaviors, not just tobacco use. It may be more appropriate to base the calculation on the prevalence of risky behaviors among college students reported in previous studies, for example using the median rate across different behaviors.

5. The inclusion criteria restrict participants to ages 18–22, but the rationale for this age range is unclear. In the introduction, the cited evidence refers to individuals under 24. Are college students in India generally under 22? If some students are older due to gaps or delayed enrollment (e.g., 22–24 years), will they be excluded? The authors should clarify the reason for this age restriction and justify why older college students are not included.

6. line 166, the authors mention that they listed 27 important behavioral risks. It is unclear what the basis for this selection is. Are these all behavioral risks included in the YRBSS, or were they selected by the authors as deemed important? If the latter, a supporting reference should be provided. If the YRBSS includes only these 27 risks, this should be clearly stated in the Study Tool section.

7. Supplementary Table S2 shows that the 27 behavioral risks are grouped into seven categories: Injury risks, Victimization, Depression and suicide risk, Substance use, Nutrition and diet, Physical activity, sedentary behavior and sleep, and Sexual behavior. The authors should describe this categorization in the Study Tool section. Without this explanation, readers may find it difficult to understand the composition and classification of the 27 important behavioral risks.

Results

8. The label for Class 2 (substance use and sexual risks) may be misleading. As shown in Supplementary File S4, Class 2 is primarily characterized by alcohol use and smoking, whereas cannabis use and tobacco use are both zero. In fact, cannabis and tobacco use are significantly higher in the Multiple Risk group. Naming Class 2 as substance use and sexual risks could therefore give a misleading impression of its behavioral profile.

9. In Class 2, 10.9% of participants reported substance use before their last intercourse, yet the data show that cannabis use and tobacco use are both zero. This appears inconsistent. If substance use before sexual activity does not include cannabis and tobacco, the assessment of substance use may be incomplete, potentially overlooking important behaviors.
